# OLDER MIGRANTS IN THEIR SOCIAL CONTEXTS

**DOI:** 10.1093/geroni/igae098.1672

**Published:** 2024-12-31

**Authors:** Ashley Ritter, Allen Glicksman, Freya Diederich

**Affiliations:** Chair; Co-Chair; Discussant

## Abstract

It is recognized that a comprehensive understanding of the experiences and circumstances of older adults necessitates an examination of the broader social, institutional, and political frameworks within which they reside. The migration of older individuals entails a transformation in their relationships with family members, social networks, service providers, and governmental institutions. Lawton’s Environmental Press model, which posits that the influence of environmental factors on health and well-being intensifies as individuals age and become more fragile, can be extended to older migrants. Such individuals may find themselves relying more heavily on familial support, social networks, formal caregiving services, and governmental assistance to maintain their health compared to their native environments. The four papers presented in this panel explore the challenges confronting older migrants within these expansive contexts, probing the ways in which these contexts can either positively or negatively impact the well-being of older adults. The papers progress from an examination of familial dynamics (Reyes) to the broader societal milieu (Torres), followed by an analysis of formal care systems (Glicksman et al.), and concluding with an investigation of governmental policies at the national level (Kilkey). Collectively, these papers underscore the significance of social, institutional, and governmental frameworks in shaping the health and well-being outcomes of older migrants. Moreover, the papers underscore the necessity of employing diverse research methodologies, including surveys, qualitative inquiries, secondary data analyses, and literature reviews, to address the multifaceted nature of these issues and to cater to the specific inquiries at hand. International Aging and Migration Interest Group Sponsored Symposium

